# Design of Optimally Constructed Metabolic Networks of Minimal Functionality

**DOI:** 10.1371/journal.pone.0092583

**Published:** 2014-03-25

**Authors:** David E. Ruckerbauer, Christian Jungreuthmayer, Jürgen Zanghellini

**Affiliations:** 1 Austrian Centre of Industrial Biotechnology, Vienna, Austria, European Union; 2 Department of Biotechnology, University of Natural Resources and Life Sciences, Vienna, Austria, European Union; Koc University, Turkey

## Abstract

**Background:**

Metabolic engineering aims to design microorganisms that will generate a product of interest at high yield. Thus, a variety of *in silico* modeling strategies has been applied successfully, including the concepts of elementary flux modes (EFMs) and constrained minimal cut sets (cMCSs). The EFMs (minimal, steady state pathways through the system) can be calculated given a metabolic model. cMCSs are sets of reaction deletions in such a network that will allow desired pathways to survive and disable undesired ones (e.g., those with low product secretion or low growth rates). Grouping the modes into desired and undesired categories had to be done manually until now.

**Results:**

Although the optimal solution for a given set of pathways will always be found with the currently available tools, manual selection may lead to a sub-optimal solution with respect to a metabolic engineering target. A small change in the selection of modes can reduce the number of necessary deletions while only slightly reducing production. Based on our recently introduced formulation of cut set calculations using binary linear programming, we suggest an algorithm that does not require manual selection of the desired pathways.

**Conclusions:**

We demonstrated the principle of our algorithm with the help of a small toy network and applied it to a model of *E. coli* using different design objectives. Furthermore we validated our method by reproducing previously obtained results without requiring manual grouping of modes.

## Introduction

Microorganisms are increasingly used as cell factories to produce a multitude of chemicals and promise great potential for many future applications [1]–[4]. Microorganisms provide many benefits as production hosts: (i) production of substances that are basically inaccessible to classical chemical synthesis (e.g., proteins with specific glycosylation patterns), (ii) production of substances in a cheaper and more environment friendly way (cheap educts, processes at room temperature, no need for heavy metal catalysts, fewer by-products), and (iii) production of bulk chemicals from renewable resources instead of petroleum-based feedstocks. A multitude of (genetically engineered) microorganisms are currently used in industry. There are a few examples where microorganisms naturally produce a product of interest with sufficient yield, but it is generally necessary to genetically engineer strains to obtain the desired properties. These genetic interventions may lead to optimized channeling of metabolic fluxes towards the product of interest and/or introduce non-native pathways to enable production of foreign components [5]–[7]. Designing such strains may occasionally be straightforward, such as overexpressing a gene involved in the pathway leading to the desired product. However, multiple, non-intuitive genetic interventions are possible and often more effective due to the high connectivity of metabolites (particularly in redox- and energy-metabolism). Thus, a systems biological analysis approach is needed that considers the metabolic network structure.

Methods based on constraint-based reconstruction and analysis (COBRA) have been used successfully to predict complex intervention strategies in metabolic engineering. COBRA methods analyze the steady state behavior of an organism using its stoichiometric matrix as the main input. The stoichiometric matrix is a comprehensive, organism-specific collection of the stoichiometry of the biochemical reactions occurring in the organism of interest. Frameworks such as OptKnock [8] and RobustKnock [9] allow predicting genetic interventions that optimize host production capabilities. Both methods couple a biologically motivated objective (typically maximization of biomass production) with an engineering objective (e.g., maximize product yield). As these methods rely on an optimization principle to describe cellular behavior, they are considered biased [10].

Alternatively, elementary flux mode (EFM) analysis [11], [12] can be used to provide an unbiased view on the steady state capabilities of an organism [13]. An EFM is a minimal steady state pathway through a metabolic network [14], [15]. Minimal in this context means that removing any one reaction participating in the pathway will block any steady state flux through it. Calculating the EFMs is computationally expensive [16], as their number increases exponentially with the number of reactions and is currently limited to small and medium-scale models [17]. Such models may already lead to several hundred million EFMs, but their size is sufficient to describe core metabolism (glycolysis, pentose phosphate pathway and citrate cycle) and pathways involved with the product of interest.

One important property of EFMs is that every feasible flux distribution in the network can be described as a non-negative, linear combination of EFMs. This suggests that the entire metabolic space of the system can be represented by the full set of EFMs. Consequently, an optimal production host can be designed if EFMs with unfavorable properties, e.g., low productivity, are removed, while favorable modes with high product yield are maintained. An undesirable EFM can be removed easily if one of its participating reactions is deleted. Notice that this strategy does not rely on any biologically motivated objective in contrast to OptKnock [8] and RobustKnock [9] but only utilizes an engineering objective: Find a set of reaction deletions that will restrict the cell to desirable metabolic states only.

The concept of removing unwanted modes under the condition that certain modes have to “survive” the intervention is called constrained minimal cut sets (cMCSs) [18]. When the EFMs are known and classified into desirable and undesirable modes, not only one but all possible MCSs leading to a desired state can be calculated, which usually offers different options for biological implementation. Successful examples include [12], [19]–[22].

In this study, we address the tedious necessity of manually selecting modes that should be kept or disabled. cMCSs are dependent on allocation of the modes, and it is possible that a “better” design (e.g., with fewer deletions) could be found if only the allocation of the modes is just slightly changed (possibly leading to marginally worse production). Here we present the formulation of a binary integer program (BIP) for calculating cMCSs such that manual mode selection is no longer necessary.

## Methods

### Introductory Example

**Figure 1:**
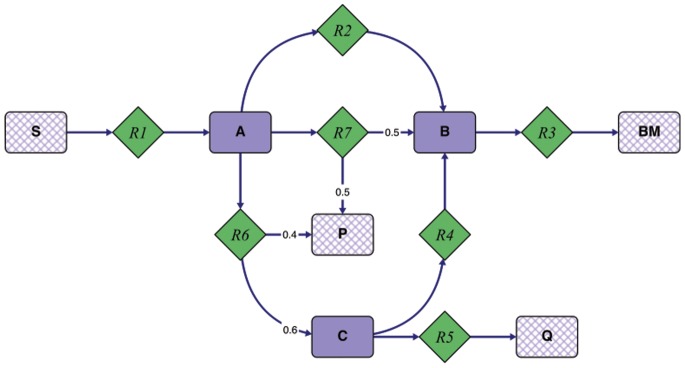
Simple metabolic toy network. The network consists of seven metabolites and seven irreversible reactions. We assume that metabolites A, B, and C are in steady state. Metabolites S, BM, P, and Q are not subject to the steady state assumption, as they are external metabolites.

**Figure 2:**
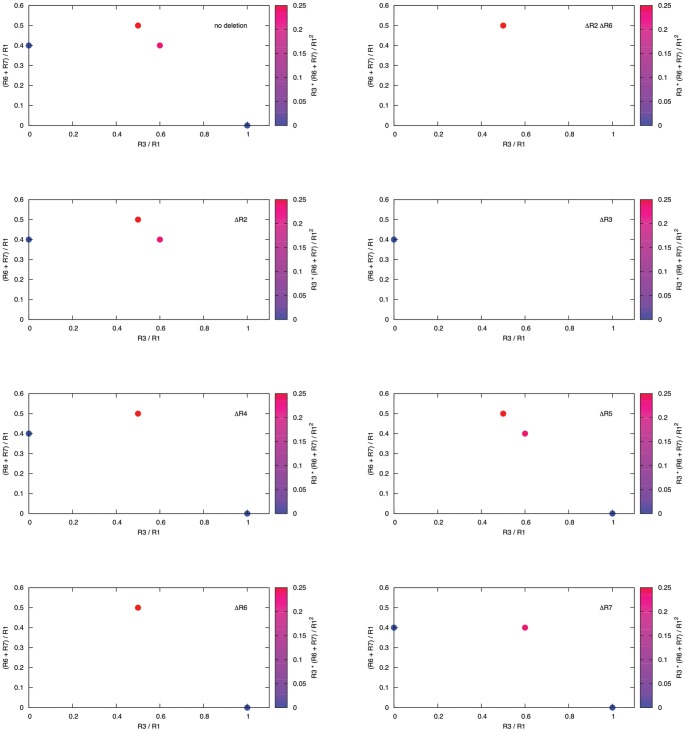
Phenotypic space of the example network in Figure 1 for different reaction deletions. The y-axis depicts the normalized product yield (R6+R7), whereas the x-axis shows the normalized specific growth rate (R3). Each dot represents an elementary flux mode (EFM). EFMs are color coded with respect to 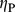. The top left panel shows the available space for the unperturbed network. The top right panel shows the “optimal” phenotypic space where only the mode with the highest product yield for the production of P (EFM3, see Table 1) is present. Such a design can be realized by deleting at least R2 and R6 or R2, R4, and R5. The remaining panels show different phenotypic spaces for different single reaction deletions (see label in each panel).

Consider the system depicted in Figure 1. The network utilizes the substrate S to produce biomass (BM), a product of interest (P), and a by-product (Q). We define the product yield, 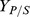 as rate of product formation per substrate uptake rate. Furthermore we define the substrate-specific productivity (SSP) as product yield times specific growth rate (biomass flux/substrate uptake flux) [23]. We are interested in finding a genetic intervention strategy which allows efficient production of P. To identify desirable network states we performed an EFM analysis on the network. The EFMs are listed in Table 1. EFM3 exhibits the maximum product yield, 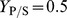. To optimize production we therefore considered all other modes to be undesirable as they have smaller product yields (see Table 1 and top left panel in Figure 2). Based on this categorization we set up an intervention which disables all undesirable functionality in the network (i.e. EFM1, EFM2, and EFM4) while keeping EFM3 operational. The desired design requires at least two deletions (R2 and R6). Additionally, a second MCS with three deletions (R2, R4, and R5) results in the same design. However, we may ask if it is possible to reduce the number of deletions if we (slightly) change the partitioning of the EFMs. If so, what is the best way to re-categorize the EFMs? For example, if we considered EFM2, EFM3, and EFM4 to be desirable (as their 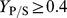) and only EFM1 to be undesirable (as it does not produce any product), we would require only a single deletion (R2, see Figure 2). Alternatively, we may ask what is the “best” achievable design with just one deletion. Here, best is meant with respect to our design objective, e.g., maximizing the minimal product yield 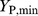.

**Table 1:** Elementary flux modes for the example network in Figure 1.

EFM	R1	R2	R3	R4	R5	R6	R7	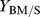	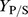	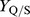	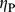
1	1.0	1.0	1.0	0.0	0.0	0.0	0.0	1.0	0.0	0.0	0.00
2	1.0	0.0	0.6	0.6	0.0	1.0	0.0	0.6	0.4	0.0	0.24
3	1.0	0.0	0.5	0.0	0.0	0.0	1.0	0.5	0.5	0.0	0.25
4	1.0	0.0	0.0	0.0	0.6	1.0	0.0	0.0	0.4	0.6	0.00

Depicted are the product yield 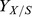 for biomass (BM), product (P), and side product (Q) as well as the mode’s substrate specific productivity, 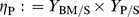, for each mode.

### Theory

We have utilized a BIP to calculate cMCSs [24]. In this formulation an EFM 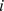 is represented by a binary vector 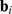. (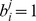 if there is a flux through the 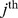 reaction of mode 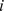, otherwise 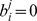.) Similarly, a cut set is represented by the binary vector 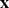. (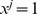 means that reaction 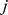 is not affected, whereas zero means it is knocked out.) To check if an EFM is hit by a cut set, we calculated the dot-product between 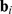 and 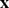. If 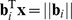 then EFM 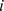 is not cut by 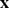 as none of the reactions contributing to EFM 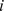 is affected [24]. If a cut set hits EFM 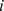 then 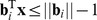. In this case EFM 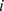 is removed from the metabolic capabilities of the network due to the property of minimality. Only one contributing reaction needs to be deleted to render a steady state flux through this mode infeasible. These two conditions can be used to set up an optimization problem, where 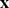 is maximized in such a way that all desired EFMs obey the former condition, while all undesired EFMs are subject to the latter constraint [24]. This approach requires a manual partition of the EFMs into desirable and undesirable modes.

To avoid this manual partitioning we assign each EFM 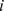 a weight 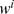. For example, we can use the product yield of each mode as its weight. In metabolic engineering we are interested in maximum product yield. To achieve this we typically couple product formation to growth. That is, we want obligatory production of the product of interest at any growth rate. Thus, similar to the RobustKnock approach [9], we search for an intervention strategy that selects modes such that the minimal yield of all modes (i.e., minimal weight of all modes) contributing to the final design will be maximized. This can be formalized mathematically in a BIP as follows:
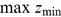
(1a)

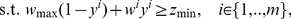
(1b)




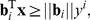
(1c)




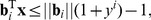
(1d)




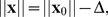
(1e)




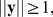
(1f)




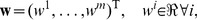
(1g)




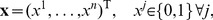
(1h)




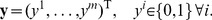
(1i)where 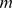 denotes the total number of modes in the unperturbed network, 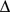 is the number of required reaction deletions and 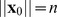 represents the total number of reactions in the system. The binary variable 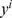 indicates whether or not EFM 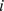 is selected for the final design. Due to the constraint (1c), EFM 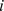 is kept if 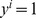. Otherwise constraint (1d) guarantees that mode 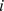 is removed. When mode 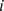 is deleted it does not contribute to the maximization problem, as constraint (1b) simplifies to 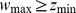, which is always an upper bound if we choose 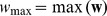. Finally, constraint (1f) requires that each design consist of at least one EFM.

We can find alternate solutions to the optimization problem equation (1) if we exclude any previous solution 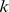 by the inequality [25].
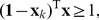
(2)where 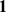 denotes an all-one vector.

The BIP in equation (1) calculates cut sets that maximize 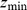 for any fixed 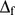. However, these cut sets may not necessarily be MCS. To obtain only the “best” MCS, we solve the system consisting of equation (1) and equation (2) repeatedly starting with 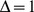. Additionally we require that at each iteration, 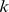, 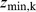 is either better or equal to the 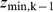 of the previous iteration 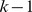. If we do not find any other solution, we increase 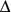 and start the cycle again until we reach the desired 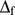. Note that equation (2) not only excludes previous solutions but also eliminates higher order cut sets (i.e., cut sets that are supersets of the already calculated MCS). Thus, this procedure only yields MCSs.

Finally, the conventional BIP formulation for cMCS [25], where modes are classified manually, is recovered if we assign a weight of one to all desired modes and a weight of zero to all undesirable modes.

To illustrate our algorithm we applied it to the toy network depicted in Figure 1 and optimized for 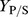. The results are depicted in Table 2.

**Table 2:** Result of our algorithm when applied to the toy model in Figure 1 (optimization for 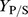).

Δ	MCS	EFMs	z
1	R2	2,3,4	0.4
1	R3	4	0.4
2	R2,R6	3	0.5
3	R2,R4,R5	3	0.5

Column 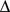 contains the number of deletions; MCS lists the deleted reaction(s) for this MCS; EFMs contains the surviving EFMs (numbers correspond to Table 1), and z is the minimal value for the product yield, 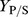, in the system.

### Implementation

To solve the BIP, we use the IBM ILOG CPLEX Optimizer, http://www.ibm.com/software/integration/optimization/cplex-optimizer, for which free academic licenses are available. We used the specialized CPLEX’ feature populate [26] to speed up the calculation of alternate optima, which allows for efficient generation of multiple solutions with one function call. Our implementation of the algorithm in C is available from the authors on request.

## Results

In the previous section we described our approach to determine cMCSs without preselecting modes. Now we apply our method to optimize anaerobic ethanol production from glucose in *E. coli* using the metabolic model by Trinh *et al.* [20]. Under these conditions, the model contains 47 metabolites and 59 reactions that give rise to 5,010 EFMs.

### Optimization for Substrate Specific Productivity (SSP)

**Figure 3:**
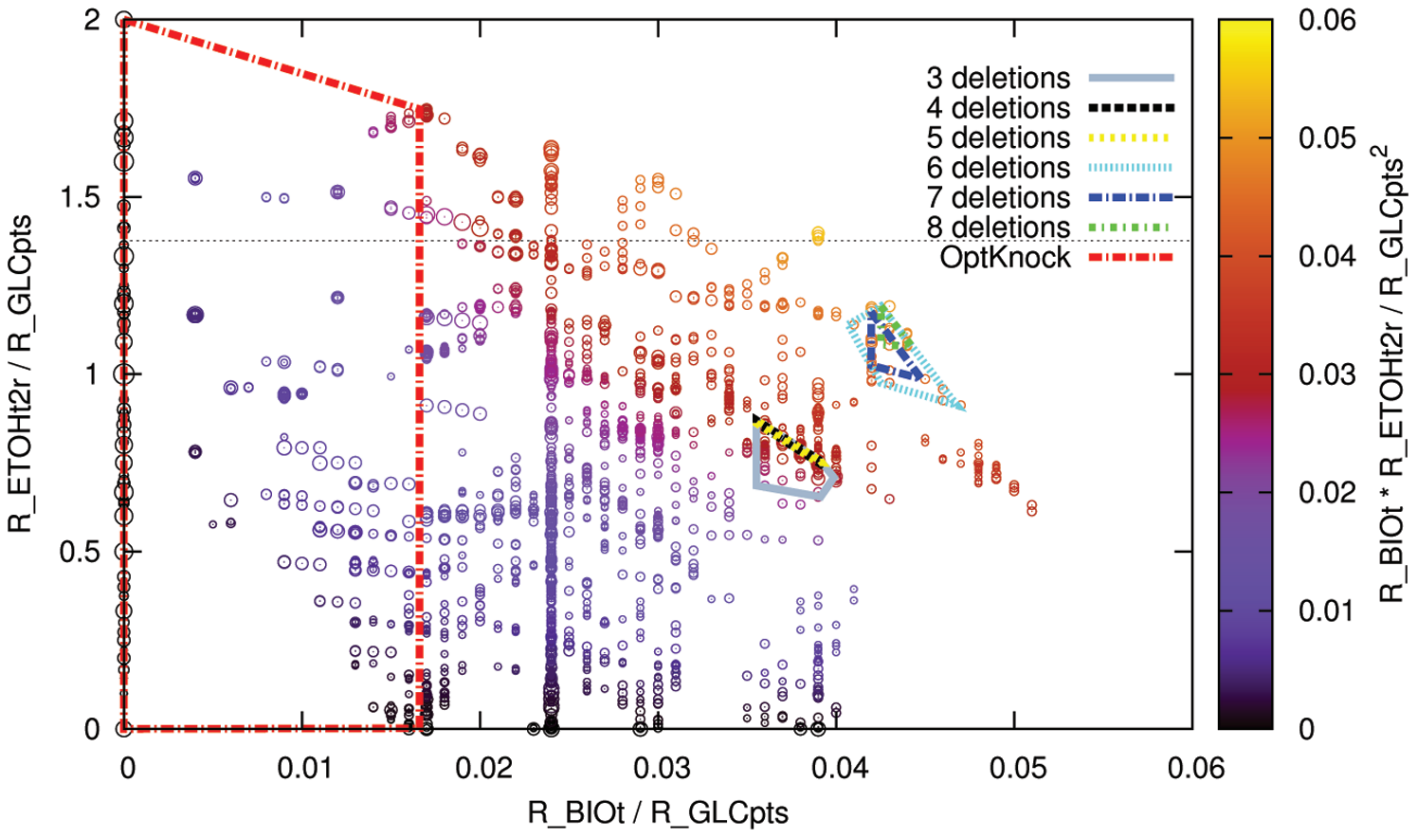
Phenotypic space for the *E. coli* model. Each circle represents one or more modes (indicated by size) with the respective flux value for ethanol secretion and biomass production, both normalized to glucose uptake. Color coding represents efficiency, defined as the product of ethanol yield (R_ETOHT2r/R_GLCpts) and specific growth rate (R_BIOt/R_GLCpts). The boxes envelop the solution space obtained for OptKnock [8] and the best solution for each deletion obtained by our algorithm (3–8 deletions). Note that while OptKnock gives different solutions for each number of deletions, the phenotypic space of these solutions remains the same.

We aimed to design the “most efficient” ethanol producing *E. coli* strain. Here, efficiency, 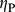, is understood to be synonymous to the SSP and defined for each EFM as the product of its ethanol yield, 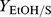, times its growth rate (biomass yield, 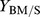), each normalized with respect to glucose-uptake. It is useful to use weights that depend on both product yield and growth rate to optimize the time-space yield of the fermentation process. We use the efficiency as weights in our analysis and maximized the minimum efficiency of the engineered *E. coli* as function of the required reaction deletions using our approach (see Table 3 and Figure 3).

**Table 3:** Results for optimization for efficiency, SSP.

			EFMs		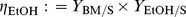		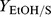		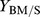	
Δ	MCSs	max 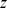	min	max	min	max	min	max	min	max
3	1	0.024368	63	63	0.024368	0.030906	0.654015	0.869893	0.035529	0.039878
4	1	0.029197	36	36	0.029197	0.030906	0.746165	0.869893	0.035529	0.039205
5	2	0.029287	33	33	0.029287	0.030906	0.750476	0.869893	0.035529	0.039205
6	32	0.041447	16	66	0.041447	0.050698	0.912292	1.192175	0.040682	0.046765
7	16	0.043016	16	16	0.043016	0.049371	0.986316	1.176466	0.041966	0.044792
8	32	0.046073	10	42	0.046073	0.050698	1.078689	1.192175	0.040682	0.044245
9	16	0.047726	12	12	0.047726	0.055150	1.078689	1.401114	0.039362	0.044245
10	240	0.055150	4	4	0.055150	0.055150	1.401114	1.401114	0.039362	0.039362
11	1968	0.055150	2	4	0.055150	0.055150	1.401114	1.401114	0.039362	0.039362
12	6848	0.055150	1	4	0.055150	0.055150	1.401114	1.401114	0.039362	0.039362
13	12544	0.055150	1	4	0.055150	0.055150	1.401114	1.401114	0.039362	0.039362
14	11776	0.055150	1	2	0.055150	0.055150	1.401114	1.401114	0.039362	0.039362
15	4352	0.055150	1	1	0.055150	0.055150	1.401114	1.401114	0.039362	0.039362

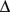
: number of deletions, MCSs: number of MCSs that lead to the same optimum, EFMs: number of EFMs surviving the intervention, 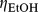, 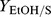, 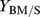: efficiency, ethanol secretion and biomass production, normalized to glucose uptake. One and two deletions do not lead to an optimum 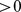, the best solution is reached with 10 deletions (no suboptimal modes survive from there on).

We found that the minimum efficiency differed from zero only for three and more deletions. That is, it is impossible to allow for up to two deletions and not to include an inefficient (i.e. 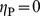) mode in the set of desired modes. Zero efficiency modes produce either no product or no biomass or neither.

At least 10 deletions are required to reach the largest possible minimum efficiency. At this stage only four EFMs with identical overall stoichiometry survive the intervention. The following solutions lead to the same optimum and only restrict the solution space further (four surviving modes to one surviving mode, see Table 3). However, the maximum number of surviving modes did not decrease with increasing cardinality of the MCSs. While the decrease can be observed as a general trend, there were two exceptions at 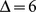 and 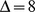. In contrast, the minimum number of surviving EFMs monotonically decreased with cardinality of the MCSs.

On our machine (2 CPUs: Intel Xeon X5650 2.67 GHz (six cores each), OS: Ubuntu 12.04) it took about 30 minutes to calculate all 37828 MCSs listed in Table 3 using 10 threads.

Figure 3 depicts a projection on biomass and ethanol flux of the total phenotypic space for the *E. coli* model used in our calculations. All 5,010 modes are represented by circles according to ethanol yield (normalized to glucose uptake) and specific growth rate (normalized to glucose uptake). Modes with identical values for both were grouped. The size of the circles corresponds to the number of modes included in these groups. The solution spaces for our method are depicted as colored polygons on the right hand side of the figure, showing the increase in minimal efficiency with each step (see also Table 3). For comparison we show the OptKnock [8] solution space (Figure 3, red dashed-dotted line). Note that although the EFM spectra may differ at each additional knockout, the overall solution space computed by OptKnock is the same for all solutions with three to eight deletions. Only the deletion of a ninth reaction leads to a state where minimal specific ethanol production is always above zero independent of growth rate.

**Figure 4:**
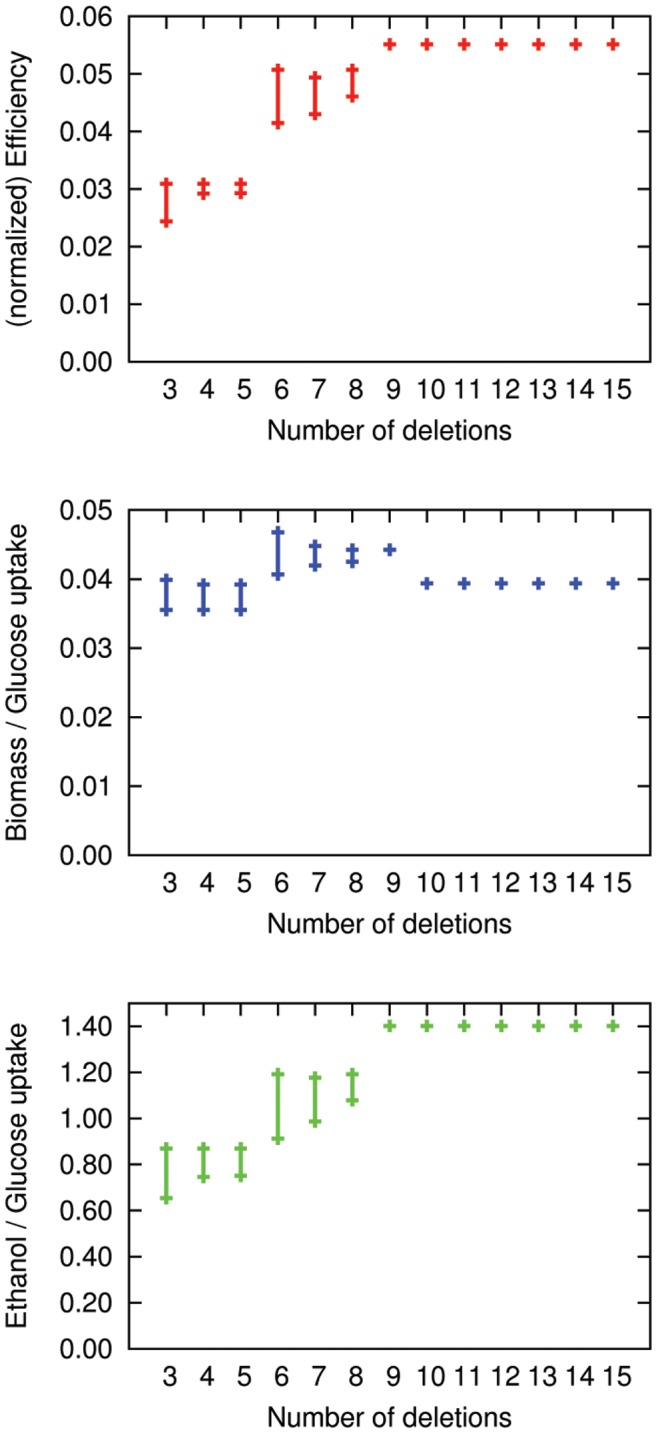
Results for efficiency optimization. Ethanol secretion per glucose uptake (lower panel), biomass production per glucose uptake (middle panel) and efficiency (ethanol/glucose times biomass/glucose) (top panel). Our algorithm shows a continuous increase in minimum efficiency with the number of deletions, but does not reach the theoretical optimum.

Figure 4 shows the span for each maximum and minimum value (ethanol flux, biomass production flux and efficiency) obtained by our algorithm. It clearly shows that the minimum efficiency calculated by our method increases with each increase in the number of deletions.

### Optimization for Efficiency with Inclusion of “Essential” Modes

We have now optimized for efficiency without any consideration of cellular maintenance. Maintenance requirements can be included in our algorithm in the form of “essential” modes. Essential EFMs are modes that must be included in the final design (either all of them or at least 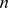 of them) independent of the objective function or any weighting. Thus, essential modes must remain unaffected by the cMCS. The enhanced algorithm can be found in Supporting Information S1.

**Figure 5:**
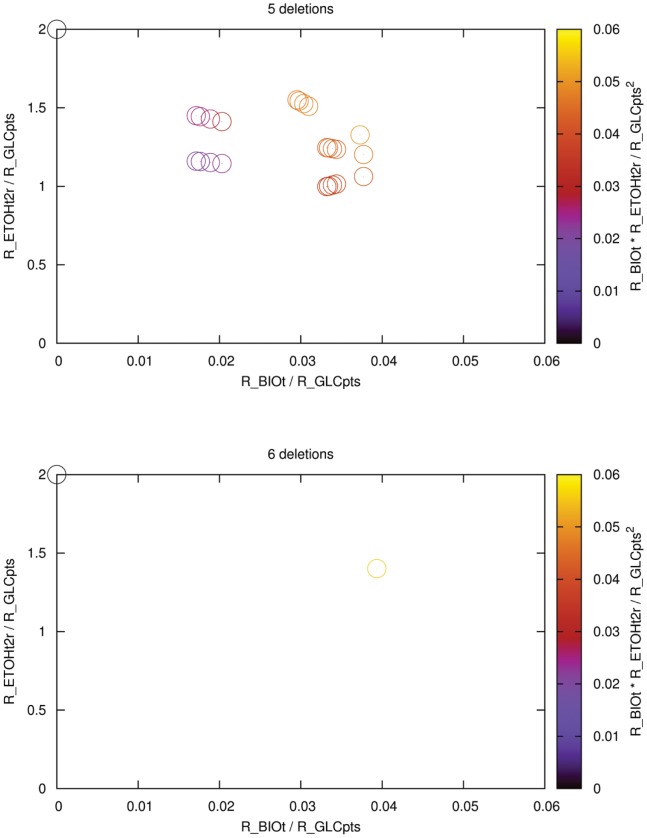
Metabolic space for optimizing the efficiency with the condition of keeping at least one “essential” mode of eight [located at (0,2)]. Best result for five deletions (top) and six deletions (bottom).

We chose the experimentally verified design used by Trinh *et al.* [20] to demonstrate the principle. The design by these authors consisted of 12 EFMs, eight of which had zero growth but maximum ethanol flux and two of the eight provided maintenance energy. We used our enhanced algorithm, optimized for efficiency and excluded those eight zero-efficiency modes from the analysis. That is, we considered those eight EFMs to be essential of which at least one had to remain in the final, engineered solution space. The smallest solution we found had five deletions (see Figure 5, top panel). We also found a solution with six deletions (see Figure 5, bottom panel), which consisted of the four most efficient modes plus all eight “essential” modes. This design corresponded to the one used by Trinh *et al.* [20]. Note that Trinh *et al.* [20] used seven deletions for their design. However, it was already pointed out in [18], [24] that the minimal number of deletions for this layout is six. Note that we manually selected the eight EFMs with the highest ethanol production (two of which produce maintenance energy) to be essential for simplicity. However, we detected all maintenance modes (or any desired subset thereof) automatically and populated the set of essential modes with them.

Importantly, we achieved the same result when optimizing for ethanol yield using equation (1) (data not shown). In that case we used the mode’s ethanol yield as weights, whereas a selection of “essential” modes was not necessary. The definition of the design objective (i.e., maximization of minimal ethanol secretion) suffices to recover Trinh *et al.*’s design.

## Discussion

cMCSs have recently been introduced to predict minimal intervention strategies for the rational design of cell factories [18], [24]. Desirable and undesirable network states are identified based on an EFM analysis, and a cMCS problem is set up, which can be efficiently solved [25]. However, the predicted MCSs will obviously depend on the categorization of the EFMs. In this report we presented a modified approach based on BIP, which avoids the necessity of grouping the modes. Instead, the selection of modes and the calculation of MCSs is automatically and optimally regulated with respect to a user defined objective. Here, we used maximizing the minimum SSP, 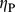, as the design criterion, among others. Maximizing the minimum of an objective is reminiscent of the RobustKnock approach [9], which aims for a strict growth coupling of byproduct formation. That is, maximizing the minimum guarantees that the product of interest is formed independent of growth rate. This optimization strategy is in contrast to OptKnock [8] and similar methods, which aim to maximize production but do not account for possible competing pathways. The different optimization strategies explain why we do not see a change in the available overall solution space of OptKnock over a wide range of the number of deletions compared to the results of our approach presented here (see Figure 3). However, our approach is indifferent to the nature of the objective. We can formulate a similar BIP using the OptKnock objective as well. However, our method is a single level optimization problem, whereas those of OptKnock and RobustKnock are bi-level optimization problems. We based our optimization on a preceding EFM analysis, which characterized the complete phenotypic space, whereas OptKnock and RobustKnock sample the phenotypic space with a second, inner optimization problem using an additional, biologically motivated objective.

The main advantage of our reformulation of the BIP in comparison to previous work [18], [24] is use of a user-defined objective, which avoids the need to manually select desired and undesirable network states. Suppose we identified 12 desirable EFMs, while the rest was undesirable (see Figure 5, bottom panel), calculated all MCS, and found that at least six deletions were required. Is it possible to further reduce the number of knockouts if we could reclassify the modes? Our approach was able to answer this question (see Figure 5, top panel). Because we optimize the partitioning of the EFMs based on a linear optimization principle to reach the optimum, we ensured that no better solution exists. However, alternate solutions may exist and can be calculated by applying equation (2). In principle we can address the same problem with the conventional cMCS formulation [18], [24].

We select modes, calculate all MCSs, reclassify the modes naively, repeat the analysis and check if the cardinality of these new MCS is smaller than the previously calculated ones. In the worst case we would have to check every possible classification of modes, which is computationally exhaustive if we use the conventional cMCS formulation. However, our reformulation achieves the same thing but is computationally more efficient. Although we only used model-intrinsic values as weights in this study (the ethanol yield and the SSP, we are free to choose any weights. Any user-defined distribution of weights can be used, for example to gradually favor a group of modes over others, without imposing a strict “desired” or “undesired” criterion. In contrast, we recover the conventional formulation by assigning binary weights to all desirable and undesirable EFMs [24]. To capture both aspects, the strict partitioning and the favoring of modes, our modified approach also allows for “essential” modes. These are desirable modes that are obligatorily included in the engineered design. Modes that provide maintenance energy could be potential candidates as essential modes in a design. Rather than using essential modes it is always an option to use a more general objective. Either way we were able to reproduce the experimentally implemented results by Trinh *et al.* [20].

Our analysis on maximizing the minimal ethanol yield revealed that total metabolic capabilities, as measured by the maximum number of surviving EFMs, did not monotonically decrease with the cardinality of the intervention set. This refutes a naïve expectation that the network’s flexibility or robustness decreases with increasing size of the intervention. Figure 4 indicates that strain improvement proceeds step-wise and does not correlate with the number of interventions. We found essentially the same optimum for three to five deletions with only small variations between the three situations. The optimum changes significantly at six deletions and remained more or less unchanged for the next two interventions.

One computational bottleneck in our method lies in the preceding EFM analysis. Currently only medium scale metabolic models can be calculated, as the number of EFMs explodes [16]. However, significant progress on the efficient calculation of EFMs has been made in recent years [17], which allows for analysis of realistic models, which have (partly) been tested experimentally [12], [19]–[21].

In summary, we have presented an alternative, BIP-based formulation for calculating cMCSs. Rather than manually identifying favorable and unfavorable network states we used an objective to guarantee optimal partitioning of the EFMs. We demonstrated that our approach remains computationally feasible for current metabolic engineering problems while adding much more flexibility to the design process.

## Supporting Information

Supporting Information S1
**Enhanced algorithm that allows for the inclusion of essential modes, with** ***D*** **, the set of desired modes,** ***T*** **, the set of undesired (target) modes,** 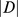 **, the number of desired modes,** 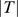 **, the number undesired modes, and n, the number of essential modes.** The general procedure is the same as explained in the text.(TEX)
